# Supplementary material for: Seminoma and Embryonal Carcinoma Footprints Identified by Analysis of Integrated Genome-Wide Epigenetic and Expression Profiles of Germ Cell Cancer Cell Lines
Source: PLoS One. 2014 Jun 2;9(6):e98330. doi: 10.1371/journal.pone.0098330 (PMC4041891; doi:10.1371/journal.pone.0098330)
Supplement: File S1 — ZIP file containing DMRforPairs output for significant regions. Please start from the html files. (ZIP) [file pone.0098330.s008.zip › figures/2215.pdf]

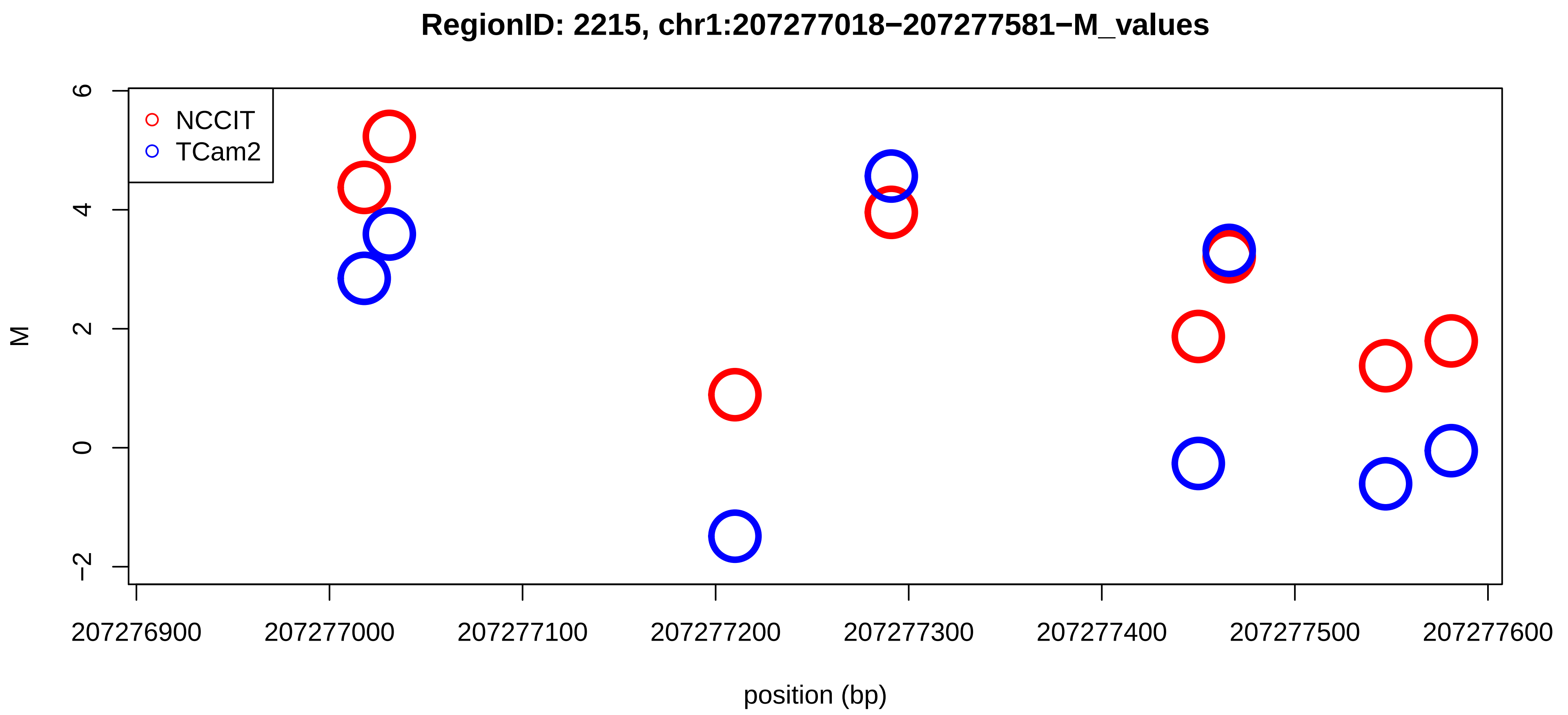

RegionID: 2215, chr1:207277018−207277581−M_values
NCCIT
TCam2
M
−2
0
2
4
6
207276900
207277000
207277100
207277200
207277300
207277400
207277500
207277600
position (bp)

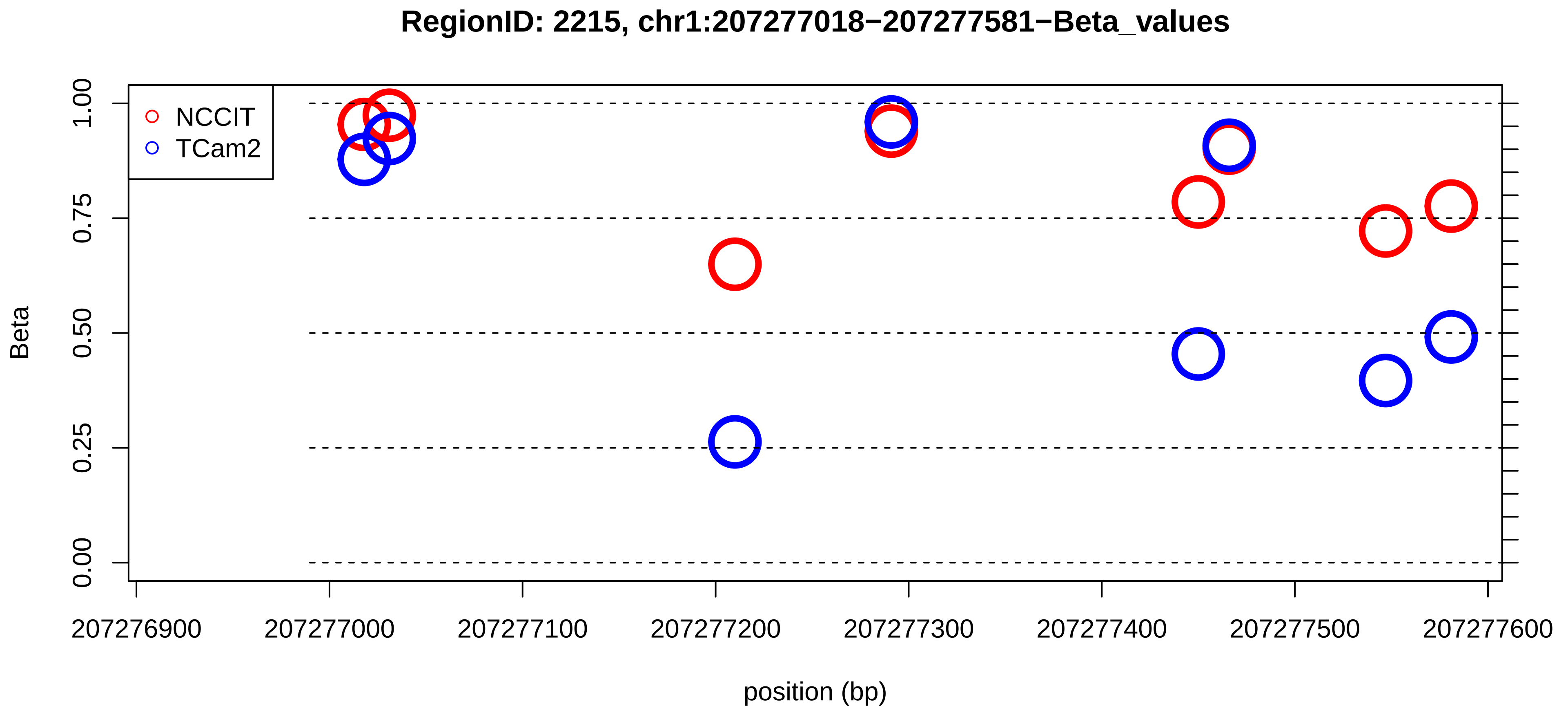

RegionID: 2215, chr1:207277018−207277581−Beta_values
NCCIT
TCam2
Beta
0.00
0.25
0.50
0.75
1.00
207276900
207277000
207277100
207277200
207277300
207277400
207277500
207277600
position (bp)
